# Implementation of the NHI (Normalized Hot Spot Indices) Algorithm on Infrared ASTER Data: Results and Future Perspectives

**DOI:** 10.3390/s21041538

**Published:** 2021-02-23

**Authors:** Giuseppe Mazzeo, Micheal S. Ramsey, Francesco Marchese, Nicola Genzano, Nicola Pergola

**Affiliations:** 1 Institute of Methodologies for Environmental Analysis (IMAA), Italian Research Council (CNR), 85050 Tito Scalo (PZ), Italy; giuseppe.mazzeo@imaa.cnr.it (G.M.); nicola.pergola@imaa.cnr.it (N.P.); 2 Department of Geology and Environmental Science, University of Pittsburgh, Pittsburgh, PA 15260, USA; mramsey@pitt.edu; 3 School of Engineering, University of Basilicata, 85100 Potenza, Italy; nicola.genzano@unibas.it

**Keywords:** volcanoes, ASTER, NHI, Google Earth Engine

## Abstract

The Normalized Hotspot Indices (NHI) tool is a Google Earth Engine (GEE)-App developed to investigate and map worldwide volcanic thermal anomalies in daylight conditions, using shortwave infrared (SWIR) and near infrared (NIR) data from the Multispectral Instrument (MSI) and the Operational Land Imager (OLI), respectively, onboard the Sentinel 2 and Landsat 8 satellites. The NHI tool offers the possibility of ingesting data from other sensors. In this direction, we tested the NHI algorithm for the first time on Advanced Spaceborne Thermal Emission and Reflection Radiometer (ASTER) data. In this study, we show the results of this preliminary implementation, achieved investigating the Kilauea (Hawaii, USA), Klyuchevskoy (Kamchatka; Russia), Shishaldin (Alaska; USA), and Telica (Nicaragua) thermal activities of March 2000–2008. We assessed the NHI detections through comparison with the ASTER Volcano Archive (AVA), the manual inspection of satellite imagery, and the information from volcanological reports. Results show that NHI integrated the AVA observations, with a percentage of unique thermal anomaly detections ranging between 8.8% (at Kilauea) and 100% (at Shishaldin). These results demonstrate the successful NHI exportability to ASTER data acquired before the failure of SWIR subsystem. The full ingestion of the ASTER data collection, available in GEE, within the NHI tool allows us to develop a suite of multi-platform satellite observations, including thermal anomaly products from Landsat Thematic Mapper (TM) and Enhanced Thematic Mapper Plus (ETM+), which could support the investigation of active volcanoes from space, complementing information from other systems.

## 1. Introduction

Several studies show the important role of satellite remote sensing in studying and monitoring active volcanoes, as a unique source of information (e.g., in remote areas where ground-based instrumentation often lacks) or as a complement to other observing systems (e.g., [1,2,3,4,5]).

Moderate-resolution Imaging Spectroradiometer (MODIS) and the Spinning Enhanced Visible and Infrared Imager (SEVIRI), and other such sensors provide Medium-Infrared (MIR; 3–5 µm) and Thermal Infrared (TIR; 8–14 µm) data at a high-temporal resolution (e.g., 15 min for SEVIRI), and are widely used to monitor thermal volcanic activity in near-real time (e.g., [6,7,8,9]). However, those sensors do not enable an accurate analysis and characterization of volcanic thermal features (e.g., lava flows; lava lakes), because of low spatial resolution (e.g., 1 km at nadir for MODIS). Advanced Spaceborne Thermal Emission and Reflection Radiometer (ASTER), aboard the Terra satellite, provides TIR data at higher spatial (90 m) and spectral (five bands) resolution, contributing to volcanological studies, despite the nominal 16 days revisit time at the equator (e.g., [10,11,12,13]). Unfortunately, ASTER Shortwave Infrared (SWIR) data, although suited to investigate high-temperature features, are no longer available due to the malfunction of SWIR detector cooling system in April 2008 (e.g., [14,15]). In order to improve the revisit time at the most active volcanoes, the Urgent Request Protocol (URP) System was developed in 2004. ASTER URP scheduling occurs following a MODIS thermal detection and the ASTER Visible and Near Infrared (VNIR) and TIR observation takes place at the next available opportunity. The URP enables an accurate analysis of different phases of thermal activity and may be used to validate thermal anomalies detected by systems using high-temporal resolution satellite data (e.g., [16,17]).

**Figure 1:**
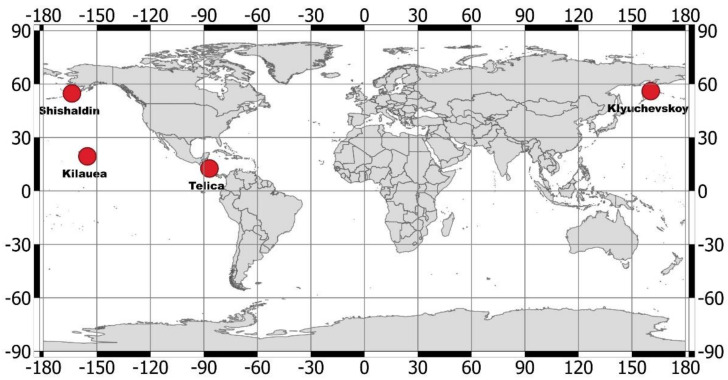
Geographic location of four active volcanoes analyzed in this work.

In this paper, we present some results of the Normalized Hotspot Indices (NHI [18]) algorithm implementation on ASTER data. The study aims to assess the NHI contribution to the analysis of thermal volcanic activity during the period 2000–2008; i.e., before the failure of the SWIR instrument. We previously tested the NHI algorithm in different volcanic areas using Multispectral Instrument (MSI) and Operational Land Imager (OLI) data [18]. The algorithm, which exhibited a good potential in mapping thermal anomalies in Thematic Mapper (TM) and Enhanced Thematic Mapper plus (ETM+) data, was then implemented within the Google Earth Engine (GEE) environment [19]. The GEE-based NHI tool enables the investigation and mapping of volcanic thermal anomalies at global scale, with low processing times, exploiting advantages of MSI and OLI data integration [19]. Here, the ingestion of the ASTER data collection available within the GEE NHI tool is also evaluated and discussed. To perform this work, we investigated the four active volcanoes of Figure 1. Those volcanoes, which are located in different geographic areas, showed intense (e.g., Kilauea; Hawaii, USA) and weak (e.g., Telica, Nicaragua) thermal activities during the period of interest (i.e., 2000–2008). We assessed the NHI detections through field reports, the manual inspection of satellite image data and comparison with the well-established ASTER Volcano Archive (AVA). The latter includes thousands of individual ASTER granules, reports, digital elevation maps and thermal anomaly products for all Holocene volcanoes (e.g., [20,21]).

## 2. Data

ASTER is a multispectral imager flying on the Terra satellite, which was launched in December 1999 in a sun-synchronous orbit at an altitude of 705 km, with a 10:30 a.m. equator crossing time. This sensor offers 14 spectral bands, ranging from visible to thermal infrared, with a high spatial and spectral resolution (see Table 1). A backward looking near-infrared band (3B) also provides along-track stereo coverage [22]. ASTER produces Level-1A (L1A), Level-1B (L1B), and various Level-2 data products, covering an area of 60 km × 60 km. ASTER L1A data are unprocessed data, containing geometric correction and radiometric calibration coefficients, which are updated periodically [22]. L1B data are generated after applying the coefficients for radiometric calibration and geometric resampling and are stored together with metadata in one Hierarchical Data Format (HDF) file. The L1B data product is generated by default in Universal Transverse Mercator (UTM) projection, using the Cubic Convolution resampling method [22]. The ASTER Level-1B data are provided in terms of scaled radiance. To convert from Digital Number (DN) to radiance *L* (W/m^2^ sr µm) at the sensor, the following formulation is used:

(1) $$L=\;\left(\mathrm{DN}\;\mathrm{value}-1\right)\;\times \mathrm{Unit}\;\mathrm{conversion}\;\mathrm{coefficient}$$

The unit conversion coefficient (UCC) (W/m^2^ sr µm) of each spectral band, which is stored in the metadata file, is detailed in Table 1. The UCC values refer to multiple different gain settings for the VNIR and SWIR data, which alter the range and amplification of the signals [23]. 

The high gain setting extends the range of DN output in the presence of a low reflectance target (e.g., dark soil/rocks), whereas the low-gain settings increase the dynamic range and the maximum radiance detectable by the sensor, reducing the saturation (e.g., [24]). The low gain-2, which was available only for the SWIR bands, was used to detect high temperature targets (e.g., lava lakes) (e.g., [17]). However, low-gain SWIR observations are less sensitive to subtle thermal anomalies and are not completely free from saturation issues (e.g., [25]). The TIR data are not equipped for multiple gains and therefore do saturate at pixel-integrated temperatures >~375 K.

## 3. Methods

### 3.1. NHI Algorithm and its Exportability to ASTER Data

**Figure 2:**
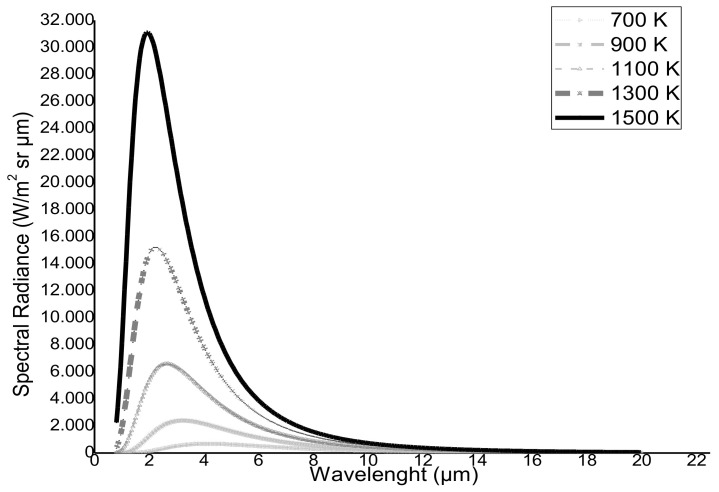
Planck curves for blackbodies at different magmatic temperatures (700–1500 K).

Several studies show that satellite sensors with channels in the SWIR band (1.4–2.5 μm) may be used to identify and quantify high-temperature features (e.g., [26,27]). Figure 2 displays the Planck curves for blackbodies at different magmatic temperatures (i.e., between 700 and 1500 K). The peak emitted radiance shifts from MIR to SWIR wavelengths with increasing temperatures.

The radiance measured by the satellite depends on several contributions (e.g., radiance emitted by the background, path radiance, and solar reflectance). The reflected solar radiance significantly affects the SWIR observations (this component is even stronger at the VNIR wavelengths), and, therefore, hot targets are generally more difficult to detect and quantify in daylight than nighttime conditions (e.g., [28]). The NHI algorithm combines two normalized indices to identify volcanic thermal anomalies in daytime by means of mid-high spatial resolution satellite data:

(2) $$NHI_{SWIR}=\frac{L_{\mathrm{SWIR}2\;}-L_{\mathrm{SWIR}1\;}}{L_{\mathrm{SWIR}2\;}+L_{\mathrm{SWIR}1\;}}$$

(3) $$NHI_{SWNIR}=\frac{L_{\mathrm{SWIR}1\;}-L_{\mathrm{NIR}\;}}{L_{\mathrm{SWIR}1\;}+L_{\mathrm{NIR}\;}}$$

In Equation (2), $L_{\mathrm{SWIR}1\;}$ and $L_{\mathrm{SWIR}2\;}$ are the Top of the Atmosphere (TOA) radiance data (W m^−2^ sr^−1^ µm^−1^) measured at around 1.6 µm and 2.2 µm. In Equation (3), $L_{\mathrm{NIR}\;}$ is the TOA radiance data measured in the NIR band, at around 0.8 µm. The $NHI_{SWIR}$ index produces positive values in the presence of hot magmatic features, due to the higher emitted radiance at 2.2 µm than 1.6 µm (see Figure 2), and negative values over background surfaces and clouds, because of reflected solar radiance [18]. However, as observed using OLI/MSI data, the limited dynamic range of the SWIR2 channel makes the $NHI_{SWIR}$ index less suited to identify intense thermal anomalies (i.e., high-temperature targets that occupy a larger portion of the pixel area). The $NHI_{SWNIR}$ index increases to positive values in areas affected by strong volcanic thermal emissions, and remains negative elsewhere, due to reflected solar radiance in the NIR band. This index enables a better identification of thermal anomalies saturating the SWIR2 channel [18]. Thus, the NHI algorithm considers pixels as “hot” if they have values of $NHI_{SWIR}>0$ OR $NHI_{SWNIR}>0$. It is capable, therefore, of successfully discriminating thermal anomalies from other targets, in spite of some limitations [18,19]. In particular, the algorithm should not detect thermal anomalies of a mid-low temperature (i.e., below 500 K [28]), which are less radiant in the SWIR band. Missed detections may also occur in presence of hot targets under clouds/plumes. In addition, NHI may underestimate intense lava flows yielding the saturation of SWIR channels [18]. To overcome this limitation, and to minimize false positives ascribable to differences in the minimum radiance in the spectral bands used, we added a spectral test for “extreme” pixels and an initial test on SWIR radiances within the tool [19]. 

**Figure 3:**
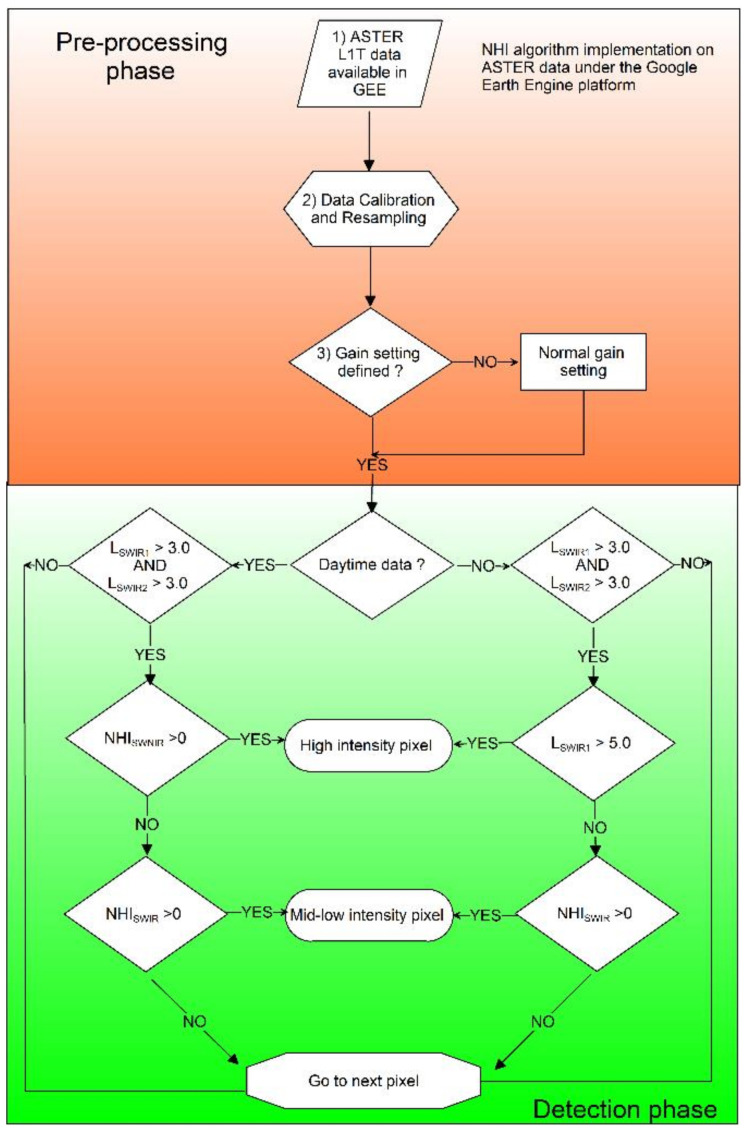
Flowchart of the Normalized Hot Spot Indices (NHI) detection scheme applied to ASTER data of 2000–2008 under Google Earth Engine (GEE).

In this work, for the first time we exported the NHI algorithm to ASTER data. Specifically, we analyzed the ASTER L1T (Precision Terrain Corrected Registered At-Sensor Radiance Product) dataset available in GEE [29]. The flowchart of Figure 3 describes the main steps of the NHI algorithm implementation, with the pre-processing phase including:

1. Conversion from DN to radiance in bands B4 (SWIR1), B5 (SWIR2) and B3N (NIR);
2. Data re-projection and resampling, using the nearest neighbor method, to analyze NIR/SWIR data at the same spatial resolution (30 m);
3. Gain setting check (see Section 4).

The detection phase, working pixel by pixel, includes an initial test on SWIR radiance (i.e., *L_SWIR1_* > 3.0 *AND L_SWIR2_* > 3.0) and the use of a different algorithm configuration to identify hot pixels in daytime and nighttime (i.e., *L_SWIR1_* > 5.0 *OR NHI_SWNIR_* > 0) conditions. 

### 3.2. The AVA Database

The AVA database is a large archive of web-accessible volcano image data, including information about spectral signatures, volcanic emissions (e.g., eruption columns and plumes), surficial deposits (e.g., lava flows), and eruption precursor phenomena (e.g., [23]). AVA provides a prompt access to hundreds of full resolution day and night ASTER scenes acquired over more than 1500 active volcanic areas, corresponding to those listed by the Global Volcanism Program (GVP). The database was continuously updated until late 2017, by ingesting full spatial resolution ASTER and Global Digital Elevation Model (GDEM) data (e.g., [30]). It provides information about volcanic thermal anomalies in terms of flagged pixels (in JPG. and TIFF. formats) and temperatures, derived from ASTER L1B granules. Thermal anomaly products from the AVA database were also used to validate hot spot detections from high-temporal resolution satellite data (e.g., [31]).

## 4. Results

In this section, we show the results of the Kilauea (HI, USA), Klyuchevskoy (Kamchatka, Russia), Shishaldin (AK, USA), and Telica (Nicaragua) investigations performed under the GEE environment using both daytime and nighttime ASTER data from March 2000 to 2008. We used a different distance buffer (DB) as input, to take into account the different size of analyzed volcanic areas (e.g., [19]). The thermal anomaly maps shown here display hot pixels in two different colors, providing qualitative information about areas where volcanic thermal emissions were strong (yellow pixels) or less intense (red pixels). 

### 4.1. Analysis of Kilauea Thermal Activity 

**Figure 4:**
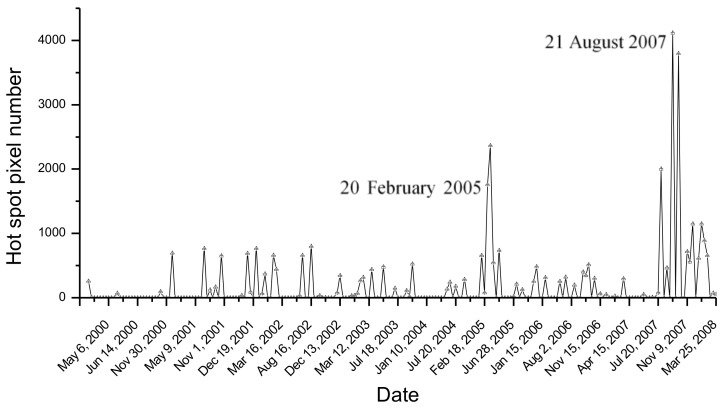
Temporal trend of hot pixels flagged by NHI at Kilauea (Hawaii, USA) volcano, by analyzing ASTER data of March 2000–2008.

Kilauea is one of the most active volcanoes in the world, and one of the first volcanoes investigated by satellite (see [32] and references herein). Since January 1983 (until 2018), Kilauea was in an almost continuous eruption, with open lava lakes and flows from the summit caldera and the East Rift Zone (ERZ), and with the emission of sulfur dioxide affecting public health, agriculture, and infrastructures [32,33]. The stop of the ERZ eruptive episode, recorded in early September 2018, marked the end of about 36 years of eruptive activity (e.g., [33]). New activity at the summit caldera resumed in December 2020. Figure 4 displays the temporal trend of hot pixels flagged by NHI at Kilauea, by setting the same DB value (50 km) used in a previous study [19]. According to NHI, volcanic thermal anomalies were particularly prevalent during February–March 2005 and July–August 2007 (see high number of hot pixels), indicating the occurrence of more intense thermal activity. 

**Figure 5:**
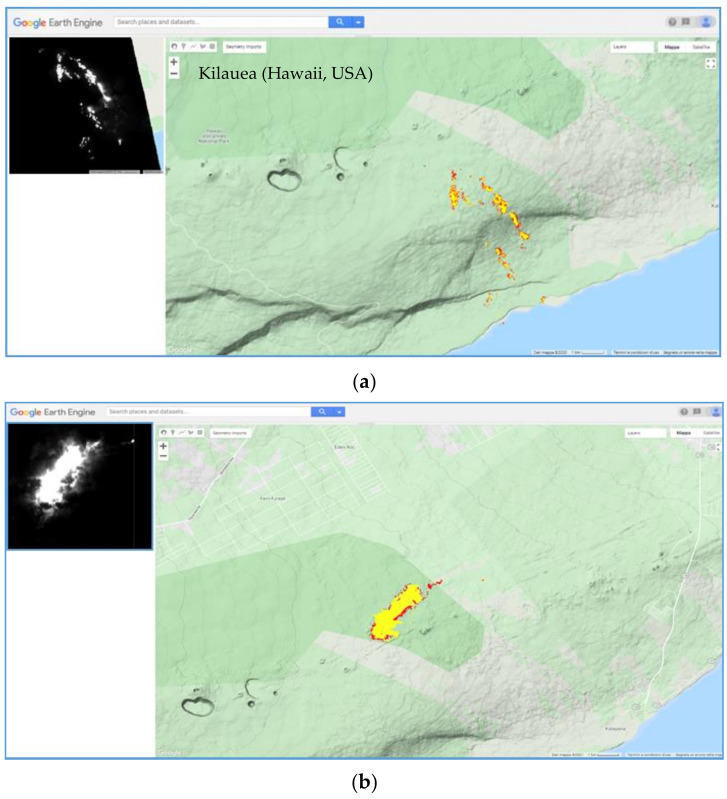
Thermal anomalies (red/yellow pixels) detected by NHI on ASTER data of March 2000–2008, overlapped onto the topographic map of Kilauea; (**a**) 20 February 2005 at 22:41 UTC; (**b**) 21 August 2007 at 22:42 UTC. On the left top, shortwave infrared (SWIR2) band 5 image whose bright pixels indicate hot features.

Figure 5 shows the thermal anomaly maps from nighttime ASTER scenes of 20 February 2005 at 08:41 UTC (22:41 LT) and of 21 August 2007 at 08:42 UTC (22:42 LT). We overlapped thermal anomalies detected by NHI to the topographic map of Kilauea volcano to retrieve accurate information about areas inundated by lava flows. In detail, Figure 5a shows that on 20 February 2005 lava flows moved in the SE direction, extending from about 2200 m elevation up to the coast (see red/yellow pixels corresponding to the most radiant ones of the SWIR2 image, displayed on top-left side of the figure). This information is in agreement with field reports, which state that lava flows were visible on the Pulama pali fault scarp and on the coastal plain, with a small stream of lava flowing from front of West Highcastle delta into the sea (e.g., [34,35]). Figure 5b displays the thermal anomaly map of 21 August 2007, showing that lava inundated the area located north of ERZ. One of the active segments of the 21 July 2007 fissure eruption, which was accompanied by the collapse of the Pu’u’Ō’ō crater, continued to feed the advancing lava flows observed by ASTER (e.g., [36,37]). 

By comparing the NHI to AVA detections, we found that the two systems marked the same phases of Kilauea activity. However, NHI considered the July–August 2007 eruption as the most intense and flagged a significantly higher number of hot pixels than AVA. Furthermore, it provided additional information about a number of thermal anomalies affecting the analyzed volcanic area, as detailed in the Discussion Section.

### 4.2. Analysis of Klyuchevskoy Thermal Activity

**Figure 6:**
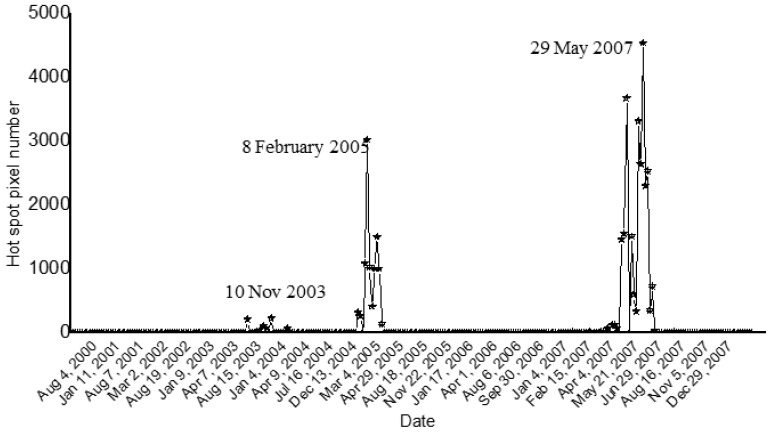
Hot pixels flagged by NHI at Klyuchevskoy (Kamchatka, Russia) by analyzing ASTER data of March 2000–2008.

Klyuchevskoy is one of the most active volcanoes of the Kamchatka Peninsula. This remote volcano has a large active crater, and frequently shows Strombolian eruptions and lava fountains [38]. Based on volcanological reports, fumarolic activity and a number of gas–ash explosions occurred at Klyuchevskoy in 2000 [39]. During 2001 and through December 2002, the volcano emitted gas and steam plumes. A Strombolian activity was recorded in August 2003, continuing through January 2004 [40,41]. Between January and March 2005, both Strombolian eruptions and lava effusions occurred. Intermittent thermal activity took place from April 2005 through January 2007, followed by the emplacement of three large lava flows beginning in mid-February 2007 and culminating in June of that year [42,43]. Independent investigations of those events were performed in previous papers [44,45]. Figure 6 displays the results of the Klyuchevskoy investigation performed setting a DB value of 8 km. In particular, the figure shows the occurrence of three main phases of thermal unrest at the target area (i.e., March 2003–January 2004; January–March 2005; January–December 2007).

**Figure 7:**
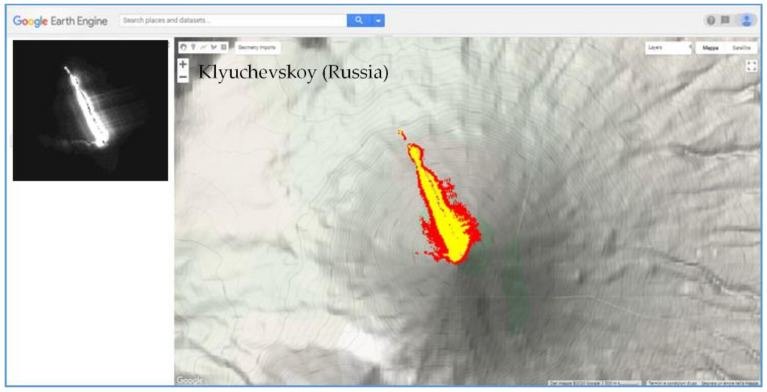
Thermal anomaly map from ASTER data of 29 May 2007 at 10:56 UTC. On the left top, shortwave infrared (SWIR2) band 5 image.

According to NHI, the eruptive phase of January–December 2007 was the most intense, and reached its peak at the end of May 2007, when a lava flow moved from the summit crater in the NW direction, reaching about 2800 m elevation, as shown in Figure 7. By comparing the above-mentioned detections to the outputs of AVA database, we found that NHI missed a number of thermal anomalies. On the other hand, as for Kilauea, it complemented the AVA observations (see the Discussion Section). 

**Figure 8:**
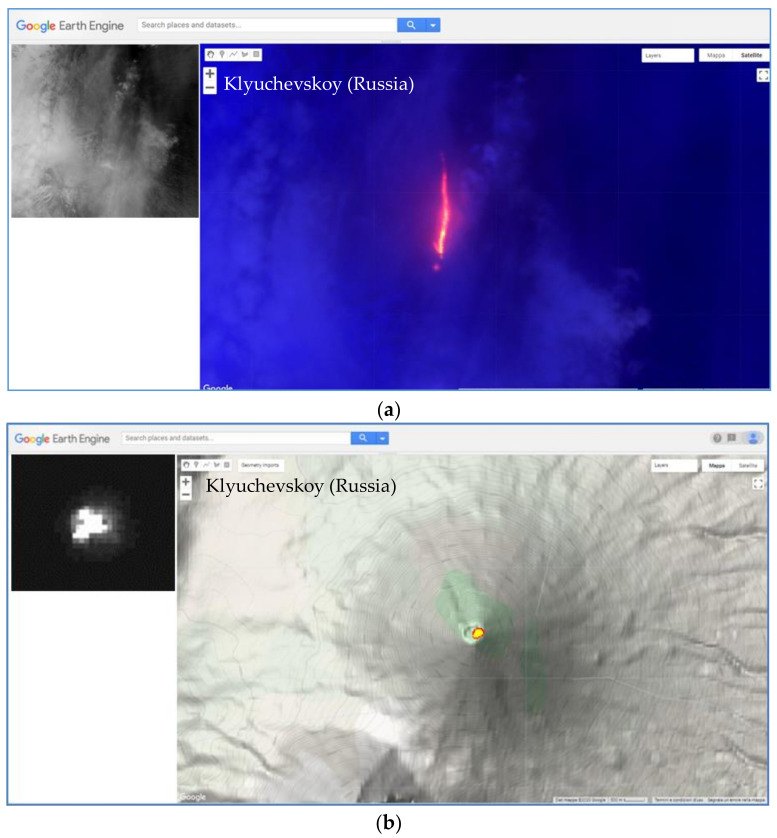
(**a**) RGB (Red = B05; Green = B04; Blue = B3N) map from ASTER data of 5 May 2007 at 00:32 UTC showing a lava flow at Klyuchevskoy undetected by NHI because of possible sub-pixel effects due to clouds (see visible image in the inset); (**b**) thermal anomaly of 4 April 2007 detected by NHI and unreported by the AVA database. On the top left of the maps, the ASTER (SWIR2) band 5 image.

Figure 8a shows an example of thermal anomaly detected only by AVA (we did not find information about the used detection scheme; see also discussion section), corroborated by the analysis of ASTER RGB (Red = B5; Green = B4, Blue = B3N) product of 5 May at 00:32 UTC (10:32 LT). The NHI algorithm did not detect the lava flow due to possible sub-pixel effects because of clouds (see visible ASTER image shown on top left side of the figure), leading to negative values of both normalized indices. Figure 8b displays an example of thermal anomaly detected by NHI, on a nighttime ASTER scene of 4 April 2007, and unreported by the AVA database. The ASTER SWIR band 5 image, displayed on the top left side of the figure, corroborates the NHI detections, confirming the capacity of the used algorithm in mapping thermal anomalies also in nighttime conditions. 

### 4.3. Identification of Thermal Anomalies at Shishaldin Volcano

**Figure 9:**
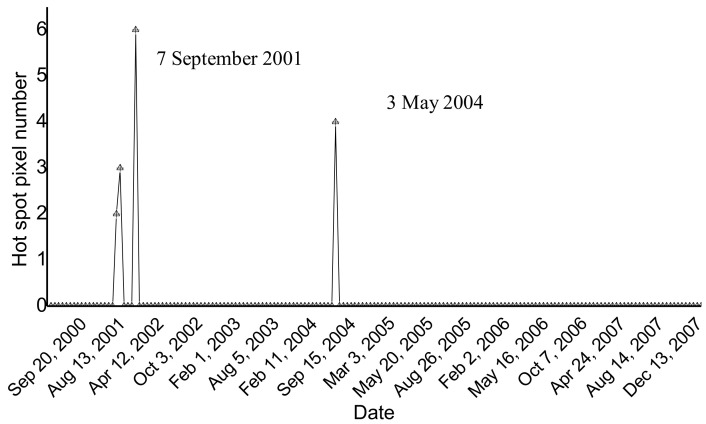
Hot pixels flagged by NHI at Shishaldin (Alaska, USA) volcano using infrared ASTER data of March 2000–2008.

Shishaldin is an active volcano located near the center of Unimak Island in Alaska, which is routinely monitored by the Alaska Volcano Observatory (AVO) using ground data and satellite observations (e.g., [46]). During the period 2000–2008, Shishaldin showed discontinuous eruptive activity, summarized in the following. In detail, after the identification of a weak thermal anomaly in August 2000, several explosions occurred the following week [47]. In mid-May 2002, the background seismicity increased; however, no thermal anomaly was visible on AVHRR or MODIS image data [48]. In August 2002, some pilots reported the emission of steam and dark clouds, whereas in early May 2004 independent satellite observations revealed the presence of thermal anomalies under optimal viewing conditions [49]. In July 2004, small ash plumes rose above the summit for at least one day [49], and in February 2008, a small ash emission probably occurred [50]. Figure 9 displays the results of the Shishaldin volcano investigation (DB = 8 km), showing the identification of thermal anomalies on a few ASTER scenes of 2000–2008. 

**Figure 10:**
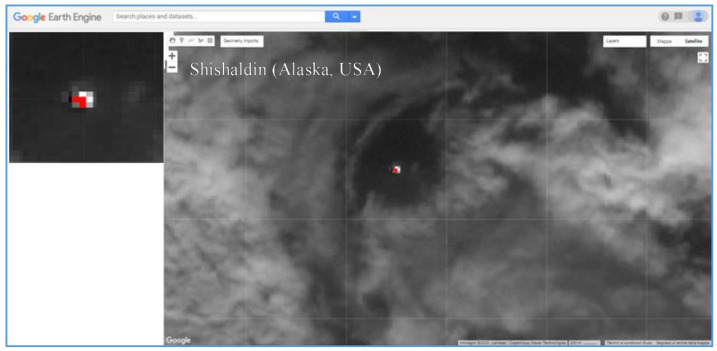
Thermal anomaly map from ASTER scene of 7 September 2001 at 22:16 UTC showing a thermal anomaly over the Shishaldin volcano. In background, the SWIR band 5 image; on top left side of the map, the detected thermal anomaly (in red).

Figure 10 shows the thermal anomaly detected on ASTER data of 7 September 2001 at 22:16 UTC (13:16 LT). Although NHI underestimated this feature due to possible sub-pixel effects, it integrated with the AVO observations, revealing the occurrence of a low-level thermal activity during June–September 2001 (see [51]). In addition, thermal anomaly identified on nighttime ASTER scene of 3 May 2004 at 08:34 UTC (23:34 LT) was in agreement with information provided by independent sources. It is worth mentioning that the AVA database did not report thermal anomalies at Shishaldin volcano before July 2014 (see [21]), as a further evidence of the relevant contribution offered by NHI in investigating thermal features in different volcanic areas.

### 4.4. Investigating Thermal Anomalies at Telica (Nicaragua) Volcano

Telica is a volcano complex located in northwestern Nicaragua, showing a persistent activity, with continuous seismicity and degassing [52]. The southern summit crater has been the source of recent eruptions, whereas fumaroles and boiling mud pots characterize the Hervideros de San Jacinto (SE of Telica), which is also site of geothermal exploration [53]. According to field reports, gas-and-ash emissions occurred at Telica volcano in early 2000 [54]. Intermittent ash explosions and crater incandescence were recorded through 2002, and strong gas emissions took place in the first half of 2003 [55]. After ash explosions and incandescence recorded in 2004, small ash explosions and a degassing activity occurred in 2005 [56]. Fumarolic activity continued through 2006, and in 2008 field observations reported the occurrence of both degassing and ash emissions [57]. 

**Figure 11:**
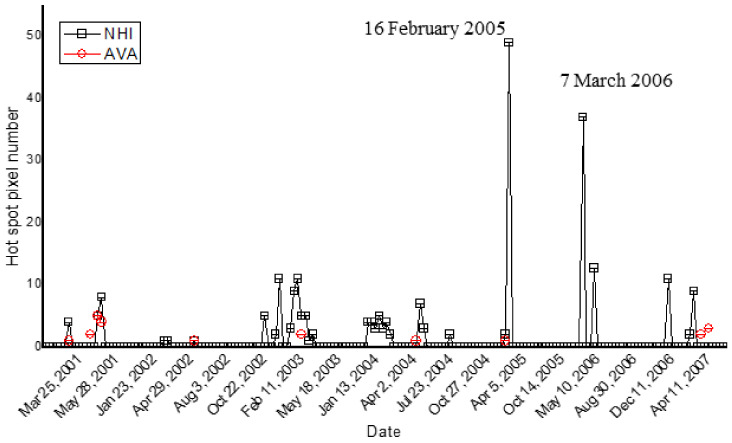
Hot pixels flagged by NHI (black squares) and AVA (red dots) at Telica (Nicaragua) volcano, from ASTER data of March 2000–2008. Note that a number of AVA detections (i.e., those of 9 March 2001; 10 April 2001, 11 February 2003; 26 March 2007; 11 April 2007) were outside the area analyzed by NHI.

Figure 11 displays the NHI (black squares; DB = 10 km) and AVA time series (red circles) retrieved at Telica volcano during the period of interest. The figure shows that NHI detected a higher number of thermal anomalies than AVA, better detailing the thermal activities of November 2002–February 2003, January–February 2004, and December 2007–March 2008. 

**Figure 12:**
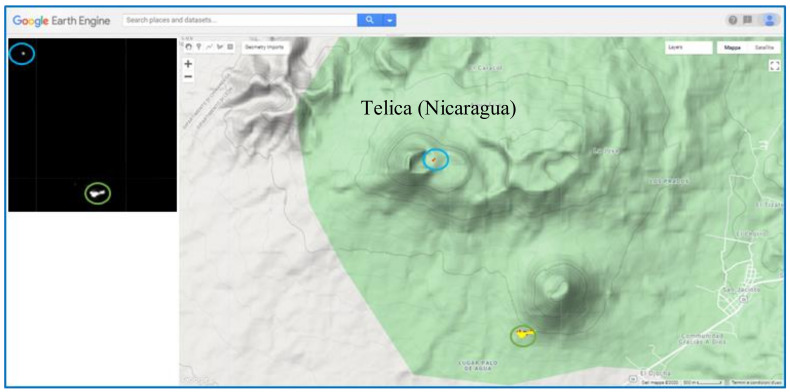
Thermal anomaly map from nighttime ASTER scene of 16 February 2005 at 04:08 UTC (22:08 LT). On top left side, the (SWIR2) band 5 image with bright pixels indicating hot targets.

The manual inspection of ASTER data confirmed the NHI detections, as shown in Figure 12 in reference to the thermal anomaly of 16 February 2005. The figure shows the identification of two clusters of hot pixels that were corroborated by the manual inspection of infrared ASTER data (see SWIR2 band 5 image). These features reveal the occurrence of thermal volcanic activity (see area marked in blue) and the possible identification of a forest fire affecting the vegetated area located SE of the crater, between 300 m and 400 m elevation (see the area marked in green). 

It is worth mentioning that some thermal anomalies in Figure 11 occurred in the surrounding of the San Jacinto-Tizate geothermal field, which is characterized by mud pools and fumaroles with temperatures around 250–285 °C [53]; these features require further investigation to be interpreted.

## 5. Discussion 

We assessed the potential of NHI algorithm in detecting and mapping volcanic thermal anomalies by means of ASTER infrared data acquired before the malfunctioning of SWIR detectors, causing saturation and severe striping [58]. 

Table 2 summarizes the results achieved comparing NHI to AVA detections. The table displays the number of ASTER images with thermal anomalies flagged by the two systems in the second and third column, and the number of unique detections (i.e., percentage of thermal anomalies identified only by NHI or AVA within the investigated areas) in the last column, in reference to the analyzed volcanic area (first column). It is worth noting that except from Kilauea, NHI flagged a higher number of thermal anomalies than AVA, which were corroborated by the manual inspection of ASTER data.

By analyzing information from Table 2, we found that a number of nighttime ASTER scenes (e.g., those of May–September 2000) did not include the SWIR bands (i.e., only TIR data were available) (e.g., [59]). Because of this issue, most thermal anomalies flagged only by AVA at Kilauea (see unique detections in Table 2) were undetectable by NHI. However, NHI did not identify volcanic thermal features when they showed low SWIR2 radiance values or produced saturation in the ASTER SWIR channels (i.e., when they were particularly weak or very intense). Some analyses are currently in progress to assess if the spectral wavelengths of ASTER, which originally provided SWIR data in six spectral channels (see Table 1), may be exploited to better detect less intense thermal anomalies (e.g., using band 9 in place of band 5 in Equation (2)).

Moreover, the use of a spectral test for “extreme” pixels, similar to that used for OLI/MSI data (see Section 3.1), is under evaluation to minimize the impact of SWIR channel saturation on NHI detections. On the other hand, the analysis of Keyhole Markup Language (KML) products made available online by AVA, revealing a pixel size of 90 m, indicated the use of TIR rather than SWIR data, explaining the significant differences in the number of hot pixels observed in comparison with NHI. The latter exploiting SWIR observations integrated the AVA detections at both Kilauea and Klyuvcheskoy, identified a number of low-level thermal anomalies at Shishaldin, and better characterized the Telica thermal activity, although some hot targets in this area probably represented fires, confirming the importance of setting a proper distance buffer within GEE, to avoid the misinterpretation of results. Hence, NHI performed well both in daytime and nighttime conditions (i.e., regardless of algorithm configuration), and even when the ASTER metadata file did not specify the UCC value for VNIR/SWIR bands. To run the algorithm in absence of this information, we analyzed the NHI outputs generated using different gain-setting modes. This analysis revealed a higher accuracy in mapping lava flows under the normal and low-gains configuration, whereas the high-gain setting mode led to the underestimation of these features. Nevertheless, since NHI also aims at mapping minor thermal anomalies, and because the low-gain 2 setting mode led to the generation of artefacts on some ASTER scenes, we assumed the normal gain when the UCC value was undefined within the header metadata file (see flowchart in Figure 3). 

These results demonstrate the successful exportability of NHI to ASTER data, despite some limitations discussed also in previous papers (e.g., [60]). This aspect is particularly relevant considering that among volcanological studies using ASTER, only some of them exploited SWIR observations to detect and characterize thermal anomalies (e.g., [15,61,62,63,64,65,66]). Moreover, the recent ASTER Volcano Thermal Output Database (AVTOD) uses TIR data to analyze volcanic thermal features over Latin America [13]. Thus, the NHI algorithm using SWIR data, which are suited to detect high-temperature targets, and showing a high confidence level of detection in both daytime and nighttime conditions, could complement information from other ASTER-based systems (e.g., AVA) over the period 2000–2008. 

## 6. Conclusions

In this paper, we assessed the potential of the NHI algorithm in detecting and mapping volcanic thermal anomalies trough daytime/nighttime ASTER data. 

By analyzing the Kilauea, Klyuchevskoy, Shishaldin and Telica volcanoes, we retrieved accurate information, in terms of location, shape, and spatial extent, about a number of volcanic thermal features, which were unreported by the AVA database. The manual inspection of satellite imagery corroborated the NHI detections, including those performed on nighttime ASTER scenes. Those detections indicate that the NHI algorithm could perform well also on nighttime Landsat-8 OLI data, whose usage could integrate daytime observations, in spite of their reduced availability. 

Although the failure of the SWIR subsystem limits the temporal range of satellite data analysis to the period 2000–2008, and despite some factors (e.g., clouds, SWIR channels saturation, gain setting modes) affecting thermal anomaly identification and mapping, this work encourages the full implementation of the NHI algorithm on ASTER data collection available in GEE. This implementation allowed us to finalize the development of a multi-platform observing system which, exploiting SWIR data from different sensors (e.g., OLI, MSI, TM and ETM+, ASTER), and, thanks to the high computational resources of the GEE platform, could successfully integrate information from other satellite-based systems. 

Finally, this study opens new scenarios for better exploitation of SWIR and NIR data, at very high spatial resolution, from new satellite missions (e.g., WorldView-3). 

## Figures and Tables

**Table 1:** Spectral and spatial features of Advanced Spaceborne Thermal Emission and Reflection Radiometer (ASTER) bands and relative unit conversion coefficient (UCC) (W/m^2^ sr µm) values, depending upon gain mode (normal, high-gain, low-gain).

Band	Spectral Range (μm)	Spatial Resolution (m)	High Gain	Normal Gain	Low Gain 1	Low Gain 2
1	0.52–0.60	15	0.676	1.688	2.25	N/A
2	0.63–0.69	15	0.708	1.415	1.89	N/A
3N	0.78–0.86	15	0.423	0.862	1.15	N/A
3B	0.78–0.86	15	0.423	0.862	1.15	N/A
4	1.60–1.70	30	0.1087	0.2174	0.290	0.290
5	2.145–2.185	30	0.0348	0.0696	0.0925	0.409
6	2.185–2.225	30	0.0313	0.0625	0.0830	0.390
7	2.235–2.285	30	0.0299	0.0597	0.0795	0.332
8	2.295–2.365	30	0.0209	0.0417	0.0556	0.245
9	2.360–2.430	30	0.0159	0.0318	0.0424	0.265
10	8.125–8.475	30	N/A	6.82 × 10^−3^	N/A	N/A
11	8.475–8.825	30	N/A	6.78 × 10^−3^	N/A	N/A
12	8.925–9.275	90	N/A	6.59 × 10^−3^	N/A	N/A
13	10.25–10.95	90	N/A	5.69 × 10^−3^	N/A	N/A
14	10.95–11.65	90	N/A	5.22 × 10^−3^	N/A	N/A

**Table 2:** NHI and ASTER Volcano Archive (AVA) thermal anomaly detections performed on ASTER data of March 2000–2008 at Kilauea, Klyuvcheskoy, Telica and Shishaldin volcanoes.

Volcano	Number of ASTER Images with Detected Thermal Anomalies		Unique Detections	
	NHI	AVA	NHI	AVA
Kilauea	80	87	8.8%	16.1%
Klyuvcheskoy	45	44	15.6%	13.,6%
Telica	37	5	86.5%	20.0%
Shishaldin	4	0	100%	00%

## Data Availability

The data presented in this study are available under the Google Earth Engine platform.

## References

[1] Harris A.J., Butterworth A.L., Carlton R.W., Downey I., Miller P., Navarro P., Rothery D.A. (1997). Low-cost volcano surveillance from space: Case studies from Etna, Krafla, Cerro Negro, Fogo, Lascar and Erebus. Bull. Volcanol..

[2] Dean K., Servilla M., Roach A., Foster B., Engle K. (1998). Satellite monitoring of remote volcanoes improves study efforts in Alaska. Eos Trans. Am. Geophys. Union.

[3] Kervyn M., Ernst G.G.J., Harris A.J.L., Belton F., Mbede E., Jacobs P. (2008). Thermal remote sensing of the low-intensity carbonatite volcanism of Oldoinyo Lengai, Tanzania. Int. J. Remote Sens..

[4] Girina O.A. (2012). On precursor of Kamchatkan volcanoes eruptions based on data from satellite monitoring. J. Volcanol. Seismol..

[5] Marchese F., Neri M., Falconieri A., Lacava T., Mazzeo G., Pergola N., Tramutoli V. (2018). The Contribution of Multi-Sensor Infrared Satellite Observations to Monitor Mt. Etna (Italy) Activity during May to August 2016. Remote Sens..

[6] Wright R., Flynn L., Garbeil H., Harris A., Pilger E. (2002). Automated volcanic eruption detection using MODIS. Remote Sens. Environ..

[7] Coppola D., Laiolo M., Cigolini C., Delle Donne D., Ripepe M. (2015). Enhanced volcanic hot-spot detection using MODIS IR data: Results from the MIROVA system. Geol. Soc. Lond. Spec. Publ..

[8] Pergola N., Coviello I., Filizzola C., Lacava T., Marchese F., Paciello R., Tramutoli V. (2015). A review of RSTVOLC, an original algorithm for automatic detection and near-real-time monitoring of volcanic hot spots from space. Geol. Soc. Lond. Spec. Publ..

[9] Gouhier M., Guéhenneux Y., Labazuy P., Cacault P., Decriem J., Rivet S. (2016). HOTVOLC: A web-based monitoring system for volcanic hot spots. Geol. Soc. Lond. Spec. Publ..

[10] Ramsey M., Dehn J. (2004). Spaceborne observations of the 2000 Bezymianny, Kamchatka eruption: The integration of high-resolution ASTER data into near real-time monitoring using AVHRR. J. Volcanol. Geotherm. Res..

[11] Pieri D., Abrams M. (2005). ASTER observations of thermal anomalies preceding the April 2003 eruption of Chikurachki volcano, Kurile Islands, Russia. Remote Sens. Environ..

[12] Reath K.A., Ramsey M.S., Dehn J., Webley P.W. (2016). Predicting eruptions from precursory activity using remote sensing data hybridization. J. Volcanol. Geotherm. Res..

[13] Reath K., Pritchard M.E., Moruzzi S., Alcott A., Coppola D., Pieri D. (2019). The AVTOD (ASTER Volcanic Thermal Output Database) Latin America archive. J. Volcanol. Geotherm. Res..

[14] Abrams M., Yamaguchi Y. (2019). Twenty years of ASTER contributions to lithologic mapping and mineral exploration. Remote Sens..

[15] Blackett M., Wooster M.J. (2011). Evaluation of SWIR-based methods for quantifying active volcano radiant emissions using NASA EOS-ASTER data. Geomat. Nat. Hazards Risk.

[16] Ramsey M.S. (2016). Synergistic use of satellite thermal detection and science: A decadal perspective using ASTER. Geol. Soc. Lond. Spec. Publ..

[17] Ramsey M.S., Flynn I.T. (2020). The Spatial and Spectral Resolution of ASTER Infrared Image Data: A Paradigm Shift in Volcanological Remote Sensing. Remote Sens..

[18] Marchese F., Genzano N., Neri M., Falconieri A., Mazzeo G., Pergola N. (2019). A Multi-Channel Algorithm for Mapping Volcanic Thermal Anomalies by Means of Sentinel-2 MSI and Landsat-8 OLI Data. Remote Sens..

[19] Genzano N., Pergola N., Marchese F. (2020). A Google Earth Engine tool to investigate, map and monitor volcanic thermal anomalies at global scale by means of mid-high spatial resolution satellite data. Remote Sens..

[20] Linick J.P., Pieri D.C., Davies A.G., Reath K., Mars J., Hubbard B.E., Sanchez R., Tan H.L. The ASTER Volcano Archive (AVA): High Spatial Resolution Global Monitoring of Volcanic Eruptions. Proceedings of the American Geophysical Union, Fall Meeting.

[21] (2020). ASTER Volcano Archive (AVA). https://ava.jpl.nasa.gov/.

[22] Abrams M., Hook S., Ramachandran B. (2002). ASTER User Handbook.

[23] Pieri D., Abrams M. (2004). ASTER watches the world’s volcanoes: A new paradigm for volcanological observations from orbit. J. Volcanol. Geotherm. Res..

[24] Yamaguchi Y., Kahle A.B., Tsu H., Kawakami T., Pniel M. (1998). Overview of advanced spaceborne thermal emission and reflection radiometer (ASTER). IEEE Trans. Geosci. Remote Sens..

[25] Wright R., Rothery D.A., Blake S., Harris A.J., Pieri D.C. (1999). Simulating the response of the EOS Terra ASTER sensor to high-temperature volcanic targets. Geophys. Res. Lett..

[26] Prakash A., Gupta R.P., Saraf A.K. (1997). A Landsat TM based comparative study of surface and subsurface fires in the Jharia coalfield, India. Int. J. Remote Sens..

[27] Layana S., Aguilera F., Rojo G., Vergara Á, Salazar P., Quispe J., Urra P., Urrutia D. (2020). Volcanic Anomalies Monitoring System (VOLCANOMS), a Low-Cost Volcanic Monitoring System Based on Landsat Images. Remote. Sens..

[28] Dennison P.E., Matheson D.S. (2001). Comparison of fire temperature and fractional area modeled from SWIR, MIR, and TIR multispectral and SWIR hyperspectral airborne data. Remote Sens. Environ..

[29] Earth Engine Data Catalogue, ASTER L1T Radiance. https://developers.google.com/earth-engine/datasets/catalog/ASTER_AST_L1T_003#description.

[30] Buongiorno M.F., Pieri D., Silvestri M. (2013). Thermal analysis of volcanoes based on 10 years of ASTER data on Mt. Etna. Thermal Infrared Remote Sensing.

[31] Lacava T., Kervyn M., Liuzzi M., Marchese F., Pergola N., Tramutoli V. (2018). Assessing performance of the RSTVOLC multi-temporal algorithm in detecting subtle hot spots at Oldoinyo Lengai (Tanzania, Africa) for comparison with MODLEN. Remote Sens..

[32] Plank S., Massimetti F., Soldati A., Hess K.U., Nolde M., Martinis S., Dingwell D.B. (2020). Estimates of lava discharge rate of 2018 Kīlauea Volcano, Hawaiʻi eruption using multi-sensor satellite and laboratory measurements. Int. J. Remote Sens..

[33] Holland L., Businger S., Elias T., Cherubini T. (2020). Two Ensemble Approaches for Forecasting Sulfur Dioxide Concentrations from Kīlauea Volcano. Weather. Forecast..

[34] USGS, Volcano Hazard Program. https://volcanoes.usgs.gov/volcanoes/kilauea/archive/multimedia/2005/Feb/main.shtml.

[35] Wunderman R., Global Volcanism Program (2006). Bulletin of the Global Volcanism Network, 31:4.

[36] Sennert S.K., Global Volcanism Program (2007). Report on Kilauea (United States). Weekly Volcanic Activity Report.

[37] Poland M., Miklius A.O., Sutton T.J., Thornber C., Wilson D. (2008). New Episodes of Volcanism at Kilauea Volcano, Hawaii. Eos Trans. AGU.

[38] Volcano Discovery, Klyuchevskoy Volcano. https://www.volcanodiscovery.com/it/klyuchevskoy.html.

[39] Wunderman R., Global Volcanism Program (2000). Bulletin of the Global Volcanism Network, 25:9.

[40] Venzke E., Global Volcanism Program (2000). Bulletin of the Global Volcanism Network, 28:11.

[41] Venzke E., Global Volcanism Program (2004). Bulletin of the Global Volcanism Network, 29:4.

[42] Wunderman R., Global Volcanism Program (2005). Bulletin of the Global Volcanism Network, 30:3.

[43] Wunderman R., Global Volcanism Program (2007). Bulletin of the Global Volcanism Network, 32:6.

[44] Rose S.R., Ramsey M.S. (2009). The 2005 eruption of Kliuchevskoi volcano: Chronology and processes derived from ASTER spaceborne and field-based data. J. Volc. Geotherm. Res..

[45] Rose S.R., Ramsey M.S., Dean K.G., Dehn J. (2015). The 2005 and 2007 eruptions of Klyuchevskoy Volcano, Russia: Behavior and effusion mechanisms. Monitoring Volcanoes in the North Pacific: Observations from Space (suppl. DVD).

[46] Lopez T., Clarisse L., Schwaiger H., Van Eaton A., Loewen M., Fee D., Lyons J., Wallace K., Searcy C., Wech A. (2020). Constraints on eruption processes and event masses for the 2016–2017 eruption of Bogoslof volcano, Alaska, through evaluation of IASI satellite SO2 masses and complementary datasets. Bull. Volcanol..

[47] Wunderman R., Global Volcanism Program (2000). Bulletin of the Global Volcanism Network, 25:8.

[48] Wunderman R., Global Volcanism Program (2002). Bulletin of the Global Volcanism Network, 27:5.

[49] Wunderman R., Global Volcanism Program (2004). Bulletin of the Global Volcanism Network, 29:6.

[50] Wunderman R., Global Volcanism Program (2008). Bulletin of the Global Volcanism Network, 33:8.

[51] Alaska Volcano Observatory (2001). https://avo.alaska.edu/volcanoes/activity.php?volcname=shishaldin&eruptionid=523&page=basic.

[52] Rodgers M., Roman D.C., Geirsson H., LaFemina P., McNutt S.R., Muñoz A., Tenorio V. (2015). Stable and unstable phases of elevated seismic activity at the persistently restless Telica Volcano, Nicaragua. J. Volcanol. Geotherm. Res..

[53] Ostapenko S.V., Spektor S.V., Netesov Y.P. (1998). San Jacinto-Tizate geothermal field, Nicaragua: Exploration and conceptual model. Geothermics.

[54] Wunderman R., Global Volcanism Program (2000). Bulletin of the Global Volcanism Network, 25:3.

[55] Wunderman R., Global Volcanism Program (2009). Bulletin of the Global Volcanism Network, 34:5.

[56] Wunderman R., Global Volcanism Program (2009). Bulletin of the Global Volcanism Network, 34:8.

[57] Venzke E., Global Volcanism Program (2018). Bulletin of the Global Volcanism Network, 43:9.

[58] Abrams M. (2000). The Advanced Spaceborne Thermal Emission and Reflection Radiometer (ASTER): Data products for the high spatial resolution imager on NASA’s Terra platform. Int. J. Remote Sens..

[59] ASTER Science Office ASTER SWIR Data Status Report. http://www.aster.jspacesystems.or.jp/en/about_aster/swir_en.pdf.

[60] Plank S., Marchese F., Genzano N. (2020). The short life of the volcanic island New Late’iki (Tonga) analyzed by multi-sensor remote sensing data. Sci. Rep..

[61] Trunk L., Bernard A. (2008). Investigating crater lake warming using ASTER thermal imagery: Case studies at Ruapehu, Poás, Kawah Ijen, and Copahué Volcanoes. J. Volcanol. Geotherm. Res..

[62] Carter A., Ramsey M. (2010). Long-term volcanic activity at Shiveluch Volcano: Nine years of ASTER spaceborne thermal infrared observations. Remote Sens..

[63] Patrick M.R., Witzke C.N. (2011). Thermal mapping of Hawaiian volcanoes with ASTER satellite data. US Geol. Surv. Sci. Investig. Rep..

[64] Gray D.M., Burton-Johnson A., Fretwell P.T. (2019). Evidence for a lava lake on Mt. Michael volcano, Saunders Island (South Sandwich Islands) from Landsat, Sentinel-2 and ASTER satellite imagery. J. Volcanol. Geotherm. Res..

[65] Lombardo V., Buongiorno M.F. (2006). Lava flow thermal analysis using three infrared bands of remote-sensing imagery: A study case from Mount Etna 2001 eruption. Remote Sens. Environ..

[66] Murphy S.W., de Souza Filho C.R., Oppenheimer C. (2011). Monitoring volcanic thermal anomalies from space: Size matters. J. Volcanol. Geotherm. Res..

